# Behind the Curtain of Abnormal Placentation in Pre-Eclampsia: From Molecular Mechanisms to Histological Hallmarks

**DOI:** 10.3390/ijms25147886

**Published:** 2024-07-18

**Authors:** Anna Gusella, Guido Martignoni, Cinzia Giacometti

**Affiliations:** 1 Pathology Unit, Department of Diagnostic Services, ULLS 6 Euganea, 35131 Padova, Italy; anna.gusella@aulss6.veneto.it; 2 Department of Pathology, Pederzoli Hospital, 37019 Peschiera del Garda, Italy; guido.martignoni@univr.it; 3 Department of Diagnostic and Public Health, Section of Pathology, University of Verona, 37129 Verona, Italy

**Keywords:** embryology, placentation, pre-eclampsia, histological hallmarks

## Abstract

Successful human pregnancy needs several highly controlled steps to guarantee an oocyte’s fertilization, the embryo’s pre-implantation development, and its subsequent implantation into the uterine wall. The subsequent placenta development ensures adequate fetal nutrition and oxygenation, with the trophoblast being the first cell lineage to differentiate during this process. The placenta sustains the growth of the fetus by providing it with oxygen and nutrients and removing waste products. It is not surprising that issues with the early development of the placenta can lead to common pregnancy disorders, such as recurrent miscarriage, fetal growth restriction, pre-eclampsia, and stillbirth. Understanding the normal development of the human placenta is essential for recognizing and contextualizing any pathological aberrations that may occur. The effects of these issues may not become apparent until later in pregnancy, during the mid or advanced stages. This review discusses the process of the embryo implantation phase, the molecular mechanisms involved, and the abnormalities in those mechanisms that are thought to contribute to the development of pre-eclampsia. The review also covers the histological hallmarks of pre-eclampsia as found during the examination of placental tissue from pre-eclampsia patients.

## 1. Introduction

Several crucial and highly controlled steps are necessary for a successful human pregnancy. Understanding the normal development of the human placenta is essential for recognizing and contextualizing any pathological aberrations that may occur [1]. Many pregnancy complications are believed to arise from problems during early placentation, particularly in cases where the trophoblast invasion is inadequate. The effects of these issues may not become apparent until later in pregnancy, during the mid or advanced stages. It is not surprising that issues with the early development of the placenta can lead to common pregnancy disorders, such as recurrent miscarriage, fetal growth restriction (FGR), pre-eclampsia (PE), and stillbirth. Furthermore, adverse pregnancy conditions can have long-term effects on the health of the fetus through developmental programming. Without the expectation of being fully exhaustive, due to the intricateness of the subject, this review covers the embryo implantation phase, the molecular mechanisms that lie underneath it, and the aberrations of the very same mechanisms that allegedly lead to the PE phenotype, as found in placental tissue from PE patients.

### 1.1. The Expected Path of Human Placentation

#### 1.1.1. Physiology of Human Placentation

**Figure 1:**
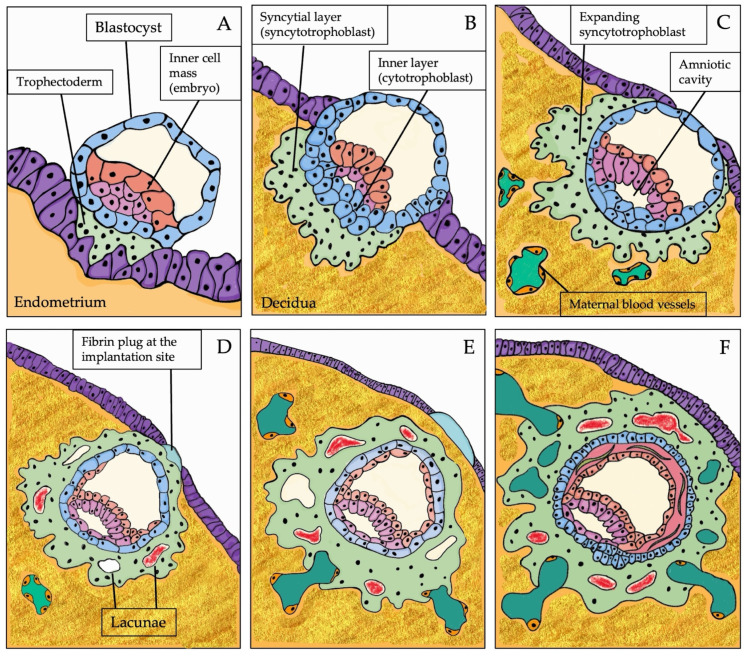
Schematic representation of embryo implantation. (**A**) Implantation at day 5–6 days POC, the blastocyst attaches to the endometrium via its embryonic pole; (**B**) at about 7–10 days POC, the blastocyst penetrates deeply in the endometrium. Two layers can be recognized: an inner layer (cytotrophoblast) and an outer syncytial layer (the syncytiotrophoblast); (**C**) the syncytiotrophoblast expands into the endometrial decidualized mucosa; (**D**) lacunae appears in the syncytiotrophoblast cellular mass; a fibrin plug covers the implantation site; (**E**) the destructive activity of the syncytiotrophoblast reaches the endometrial vessels; maternal blood flows into the lacunae; (**F**) the syncytiotrophoblast surrounds the maternal capillaries, enlarges its network of lacunae, and establishes the placenta’s circulatory system.

For the development of the human placenta, coordination between placental trophoblasts and maternal endometrium is necessary. The “normal” implantation site in the human uterus is usually in the upper part of the uterine posterior wall. Implantation is the very first stage of pregnancy, and it begins when a stable adhesion is initiated between the embryo and maternal endometrial tissue. Historically, human placentation has been classified into three steps: apposition, attachment, and invasion [2]. The apposition of the blastocyst at the uterine epithelium generally occurs about 2–4 days after the morula enters the uterine cavity [1]. At approximately 5–6 days post conception (POC), the blastocyst attaches to the endometrium via its embryonic pole, which is the place where the embryoblast adheres to the trophoblast wall of the blastocyst. During attachment, the blastocyst differentiates into an inner cell mass (which will constitute the embryo) and trophectoderm (TE), which will develop the placenta (Figure 1A). The invasion phase starts at about 7–10 days POC when the blastocyst penetrates deeply in the endometrium [3]. Two cellular layers can now be recognized: an inner layer, derived from the unfused TE, which forms the cytotrophoblast, and an outer, continuous (“syncytial”) layer, the syncytiotrophoblast, which fuses and expands to create the primary syncytiotrophoblast of the chorionic plate. Stromal endometrial cells, which surround the implanting blastocyst, differentiate and specialize into decidual cells, a process known as decidualization [4]. As the implantation progresses, within the embryonic cellular mass, the formation of the primordial amniotic cavity occurs. The syncytiotrophoblast continues its invasive activity into the maternal tissue (Figure 1B,C) [3,4,5]. After the implantation, the primary syncytiotrophoblast covers the entire gestational sac. Some irregular spaces (the “lacunae”), initially filled with uterine cell fluid and then with fetal blood, appear in the syncytiotrophoblast cellular mass. The implantation site is covered with a fibrin plug (Figure 1D). The syncytiotrophoblast grows into the uterine wall and erodes the vessel walls (capillaries, venules). The endometrial vessels connect with the fluid-filled lacunae that are now present in the syncytial mass to form a complex trabeculae pattern. This pattern facilitates the nourishment of the developing embryo with maternal blood as the blood circulates in uterine vessels and is delivered into the lacunae (Figure 1E). Starting at approximately 13–14 days POC, cytotrophoblast cells undergo proliferation and penetrate the trabeculae to form primary villi. The implantation site is fully covered by endometrial epithelium (Figure 1F). At approximately 15 days POC, the circulatory system of the placenta is almost entirely established. By the end of the third week after conception, cytotrophoblast cells permeate the primary syncytium, creating cell columns that anchor the placenta to the decidua. These cell columns spread laterally, creating the cytotrophoblast shell, a new layer between the villi and decidua. The cytotrophoblast shell first forms at the embryonic pole and then circumferentially becomes the basal plate’s precursor [2,3,6].

#### 1.1.2. The Endometrium and the Molecular Crosstalk during Implantation

Understanding the molecular events that occur at the interface between the mother and fetus is critical for ensuring successful pregnancies. It is essential to identify and characterize the diverse cell populations involved in this process and to comprehend their communication at both the molecular and cellular levels. Without the intention of being exhaustive, in this review, we try to go through that knowledge that may help to better understand pregnancy’s progression and maintenance.

Studying the first days of implantation and early embryo development is difficult because of ethical and technical issues. Most of our actual knowledge is based on histological studies performed 60–70 years ago [7] or comparative studies [8]. After fecundation, the fertilized ovum and the developing blastocyst’s nutrition is granted by the fluid secreted by the epithelium of the Fallopian tube. When the blastocyst penetrates the uterine cavity and starts the implantation process, the endometrial glands support the nutrition. Some molecules secreted by the placenta, such as human chorionic gonadotropin (hCG) and human placental lactogen (hPL), interact with receptors on the endometrial glands to stimulate glands’ secretions. Moreover, hCG stimulates the ovarian secretion of progesterone (PR), which exerts its function on endometrial glands by enhancing and maintaining their secretions and downregulating PR receptors. Once stimulated, endometrial glandular cells secrete prolactin, which maintains the secretory stimulus via an autocrine mechanism. The activation of paracrine and autocrine hormonal and signaling pathways results in the secretion of the “histotroph” [9,10,11,12,13]. “Histotroph” refers to the mix of cellular secretions, cell debris, and transudation released into the space between the maternal and fetal surfaces and is phagocyted by the trophoblast. During the early phase of pregnancy, the endometrial glands undergo a peculiar hypersecretory phenotypic modification known as the Arias-Stella reaction [14]. Endometrial glands back the developing embryo, secreting glycogen, lipid droplets, and glycoproteins (e.g., glycodelin, [15,16], mucin-1 (MUC-1) [17,18], SPP1 [19], pregnancy-specific glycoproteins [20]). During early pregnancy, the trophoblast can increase gland function and modify glycosylation. This process enables the placenta to develop in a safe and unique way. It is now clear that the endometrium plays a more significant role in promoting pregnancy and placenta formation than previously believed [13]. It is well known that the implantation mechanisms allow fetal trophoblast cells to invade and migrate into the maternal decidua [21]. Soon after the adhesion of the blastocyst to the uterine epithelium and its penetration in the decidualized mucosa, trophoblast cells invade and replace uterine spiral arteries to transform them from small, high-resistance vessels to large, low-resistance vessels. The extent and efficacy of trophoblastic invasion directly impact placental function and fetal health in late gestation. Pregnancy involves high levels of specific cytokines at the fetal–maternal interface. The implantation process regulates transcription factors, cytokines, adhesion receptors, and ligands, which affect trophoblast progenitors and uterine cells while the placenta gradually develops [22]. Leukemia inhibitory factor (LIF) is a cytokine that plays a crucial role in the early stages of pregnancy. LIF belongs to the interleukin-6 family and exerts its effects by interacting with its receptor, LIFR, and gp130. These interactions activate the Jak/STAT pathway [23,24,25,26]. LIF and LIF mRNA are expressed in the endometrium throughout the menstrual cycle. Expression increases significantly in the mid-secretory phase, which is thought to be the optimal time for implantation [27]. When analyzed, the coculture media created by Dominguez et al. showed that Interleukin-6 (IL-6) was the most abundant protein present. It was found that there was a significant difference in IL-6 levels between viable embryos that successfully implanted and those that did not. In viable embryos, more IL-6 protein was consumed in the media compared to those blastocysts that did not implant. This observation suggested that IL-6 could be vital for the final phases of the blastocyst’s development or the implantation process [28]. The link between LIF and IL-6 was observed by Ale et al., whose findings revealed that LIF and IL-6 levels were considerably higher in women who were fertile as compared to those who were infertile [29]. LIF is a strategic molecule during implantation, also for its complex interactions with different growth factors of the epithelial growth factor (EGF) family [30]. The EGF family of growth factors and their receptors regulate various biological processes, including proliferation, differentiation, and survival [31]. Heparin-binding EGF-like growth factor (HB-EGF), a member of the EGF family, utilizes various molecules as its receptors, especially ErbB1 and ErbB4 [32]. Mature HB-EGF induces dimerization of ErbB receptors, autophosphorylation, and activation of MAPK pathway [33,34]. HBEFG induces overexpression of LIF in human endometrial cells via the EGF signaling pathway [35]. While LIF upregulates the HBEGF receptor ERBB4, HEBGF signaling induces the overexpression of LIFR [36,37]. Once the trophectoderm has established contact with the uterine wall, a program of intense gene expression is initiated. Hormone production from the endometrium and ovaries plays a crucial role in ensuring the successful migration and invasion of the trophoblast and in preventing the onset of the menstrual cycle [38]. Placenta-specific transcription factors (TFs) play a significant role in developing trophoblasts by transcribing placenta-specific genes. At the beginning of pregnancy, the Yes-associated protein (YAP), the transcriptional coactivator of the Hippo signaling pathway, promotes the maintenance of trophoblast progenitor cells (TPCs—the mice equivalent of human cytotrophoblastic cells—CTB), and it stimulates the proliferation and expression of cell cycle regulators and stemness-associated genes [39,40]. Eomesodermin/T-box brain protein 2 (Eomes), caudal-type homeobox 2 (CDX2), GATA binding protein 3 (GATA3), and transcriptional enhancer factor 3 (TEAD4) are the most important TFs involved in the process of TE differentiation and development in mice [41,42]. Eomes and CDX2 play a crucial role in blastocyst development, with Eomes being overexpressed during embryo implantation. Eomes and CDX2 significantly shape and direct the blastocyst toward TE/TSC differentiation. CDX2 is important for the proper development of the TE in a pre-implantation mouse embryo and TPCs in the post-implantation phase. In the human first trimester placenta, CDX2 is also selectively expressed in the cytotrophoblast. During the pre-implantation stage, TEAD4 initiates the formation of TE by acting on CDX2 [43]. The gene expression necessary to establish the TE lineage during pre-implantation in mouse embryos is driven by TEAD4 and its cofactor, YAP1 [42,44]. The GATA family of transcription factors, highly conserved in mammals, plays a crucial role in the early development of mammalian embryos [45]. Implantation occurs in a pro-inflammatory environment, with many different inflammatory prostaglandins, chemokines, and cytokines involved. Prostaglandin E2 (PGE2) affects different stages of female fertility (e.g., meiotic maturation, ooforite cumulus expansion, follicle rupture) and also maintains the corpus luteum function to support embryo early development and implantation [46]. PGE2 production is increased in the luminal epithelium and the underlying stroma at both mice and human implantation sites, thus indicating its role in attachment and localized endometrial vascular permeability [47]. Prostaglandin E2 is considered one of the important regulators of human trophoblast invasion, which activates other signaling proteins [48,49]. As the implantation window is limited, the initial stages of implantation rely on substrate adhesion molecules produced by uterine and embryonic trophectoderm cells. To become stable, implantation needs adhesion molecules in the implanting blastocyst and the endometrial epithelium that also interact with the basement membrane. Cell adhesion molecules include cadherins, β-catenin and MUC1, desmosomes and tight junction, the immunoglobulin superfamily (e.g., I-CAM, C-CAM, N-CAM), integrins (e.g., α_v_ integrin, β_1_ integrin, and osteopontin), and selectins [50]. In the colony-stimulating factor-1 (CSF-1)-null osteopetrotic murine model, the females are infertile, as CSF-1 affects ovulation and impacts the pre-implantation embryo [51,52]. Many studies have focused on the presence of Inteleukin-1 (IL-1) during embryo implantation. IL-1 is one of the many factors that plays an important role in embryo development. The IL-1 system acts as a local regulator, influencing other systems such as matrix metalloproteinase involved in invasion and the vascular growth factors family involved in neoangiogenesis. Matrix metalloproteinases (MMPs), especially MMP-2 and MMP-9, are deeply involved in extracellular matrix remodeling during placenta villi formation and stromal invasion [53,54]. Tissue inhibitors of MMPs (TIMPs) regulate the activities of MMPs [55]. The equilibrium between MMPs and TIMPs activity is crucial in many physiological scenarios, such as angiogenesis and vascular remodeling [56].

All these findings shed light, albeit incompletely, on the intricate communication between the embryo and maternal environment. Various interconnected pathways work together during implantation and placentation to ensure a successful pregnancy.

## 2. When the Placentation Goes Wrong

The defective processes in the trophoblast differentiation, invasive potentiality, and physiological vascular remodeling may generate adverse pregnancy outcomes such as fetal growth restriction (FGR) and PE [57,58]. Fetal growth restriction is typical in PE because of uterine and placental dysfunctions and is defined as an estimated fetal weight <10th percentile for gestational age [59]. Systemic vascular resistance, impaired capillary permeability, and haemoconcentration resulting in decreased plasma volume are responsible for the origin of hypertension. The sympathetic nervous system and the renin–angiotensin–aldosterone system (RAAS) are activated to correct the relative haemoconcentration. However, this activation increases vasoconstrictors, such as thromboxane A1, and decreases vasodilators, such as prostacyclin. All this contributes even more to the increase in blood pressure [60]. At the basis of PE, numerous molecular events lead to placental dysfunction, expressed phenotypically in maternal vascular malperfusion, and its characteristic histology.

### 2.1. Molecular Markers of PE

The cause of PE is still unclear. The main hypotheses suggest that there may be disruptions in placental function during early pregnancy. An impaired spiral artery remodeling is considered an early factor, although not necessarily its primary cause [61]. Genetic predisposition seems to play a role in PE development, as a strong association has been identified between PE and gene variants involved in thrombophilia, inflammation, oxidative stress, and the renin–angiotensin system [62]. The placenta is the principal player involved in the pathogenesis of PE. This syndrome may develop from inadequate placental cytotrophoblast invasion, followed by widespread maternal endothelial dysfunction, which results in placental ischemia and hypoxia [63]. Several molecular mechanisms exist at the basis of PE, such as altered angiogenic balance, systemic inflammation, and dysregulation of the renin–angiotensin system [64,65].

#### 2.1.1. Impaired Angiogenesis

Several data suggest that alterations in circulating angiogenic factors play an important role in PE pathogenesis. Angiogenesis is the process of the development of new blood vessels from existing ones which is fundamental during placentation. In normal pregnancies, there is a balance between proangiogenic and antiangiogenic factors, which act in the invasion of the spiral arteries by the trophoblast. The balance between these two factors is lost in pathological processes such as PE. Increased levels of the antiangiogenic factors, soluble fms-like tyrosine kinase 1 (sFlt-1), and soluble Endoglin (sEng) trap circulating vascular endothelial growth factor (VEGF), placental growth factor (PlGF), and transforming growth factor β (TGFβ), respectively, decreasing their free levels, leading to endothelial dysfunction and the clinical manifestations of the disease [66]. Fms-like tyrosine kinase 1 (Flt-1), also known as vascular endothelial growth factor receptor 1 (VEGFR-1), and soluble Flt-1 (sFlt-1) are the products of *FLT-1* generated by differential mRNA processing. Excessive placental production of sFlt-1 and sEng causes their release from the placenta into the maternal circulation and contributes to hypertension, proteinuria, and endothelial cell dysfunction associated with PE [67]. Recent studies investigated the role of CD93 (complement component C1q receptor) in the migration of cytotrophoblast into uterine spiral arteries in the early phases of pregnancy. As CD93 expression seems to be independent of oxidative stress, its role could be crucial to understand the typical defective vascular remodeling of PE [68,69]. A role for complement deposition in trophoblast migration and spiral artery remodeling is supported by the evidence that pregnant C1q-deficient mice develop features of placental insufficiency and PE [70].

#### 2.1.2. Oxidative Stress

Oxidative stress is recognized to play a central role in the pathophysiology of many different disorders, including PE. The concept of a balance between pro-oxidant and antioxidant factors is pivotal in understanding the damages of oxidative stress [71,72]. Pregnancy is known to increase oxidative stress, produced by a normal systemic inflammatory response, and results in high levels of reactive oxygen species (ROS) in the body. The placenta is the main source of ROS during pregnancy. The increased oxidative stress during pregnancy could potentially cause tissue damage, to which the body (in normal situations) responds by producing more antioxidants. If oxidative stress overwhelms the antioxidant defense in the placenta, it could damage other tissues [73]. In PE, the hypoperfusion of the placenta, due to a bad invasion of the spiral arteries, causes oxidative stress, apoptosis, and trophoblastic necrosis and the consequent release of proinflammatory molecules into the blood circulation. For this reason, infarct areas, atherosclerotic lesions, and reduced perfusion are found in postpartum placentas. Placental ischemia and hypoxia result from compromised trophoblast invasion and insufficient maternal spiral artery remodeling. During a normal pregnancy, this invasion extends deeply into the spiral artery to the myometrium level, leading to extensive remodeling of the maternal spiral arterioles. In placentas that will develop PE, cytotrophoblasts cannot transition from the proliferative epithelial subtype to the invasive endothelial subtype, resulting in incomplete spiral artery remodeling. This incomplete remodeling may lead to narrowed maternal vessels and subsequent placental ischemia [74]. Oxygen and nutrients are provided to the fetus through maternal blood flow. So, if some pathologic conditions affect this flow, significant adverse effects for the fetus are produced. Placental endoglin is upregulated in PE, and sEng is released in the maternal circulation; sEng interferes with TGF-β signaling and eNOS activation and thereby causes endothelial dysfunction [75].

#### 2.1.3. Inflammation

Epidemiological data suggest that the immune system may play a role in the pathogenesis of PE [76], so it is important to consider PE as a proinflammatory state. In early pregnancy of PE, chronic inflammation is characterized by high levels of serum cytokine expression and leukocyte activation. Systemic inflammation is mediated by mechanisms like the tumor necrosis factor-alpha (TNFα), a cytokine involved in systemic inflammation. In normal pregnancy, TNF-*α* is low in the first trimester and increases with advancing gestation age [77]. It is involved in the pathogenesis of PE because it directly contributes to endothelial dysfunction [65]. Recent studies have highlighted the role mediated by the cysteine–cysteine motif chemokine ligand 20 (CCL20) among the inflammatory proteins that may display pleiotropic effects. Its level increases both in the early and late stages of PE, and it may constitute a promising biomarker in the early detection of PE since its earlier phases [78].

#### 2.1.4. Immunological Imbalance and Autoimmunity

There are multiple sites of the fetal–maternal interface, including the decidua basalis, intervillous space, and the interface between decidua parietalis and amniochorion, where immunological interaction between the mother and fetus can occur. Defective placentation, as in PE, might result from an immunological event that disrupts the maternal–fetal immune tolerance. Natural killer (NK) cells, dendritic cells, and macrophages are mediators of innate immunity. The last two are the major antigen-presenting cells in the uterus, and they facilitate adaptation of the immune response to prevent rejection of the embryo. Several studies have found a statistically significant increase in macrophages and dendritic cells in pre=eclamptic placentas compared to placentas from normal pregnancies [79]. Uterine NK (uNK) cells play a significant role in placentation by regulating the invasion of trophoblast cells into the decidua basalis and remodeling of the spiral arteries. This is due to the high abundance of uNK cells in the decidua during the first trimester. uNK cells impact placentation through cytotoxicity, local cytokine production, and trophoblast apoptosis [80]. Decidual NK (dNK) cells might be involved in developing PE. Zhang et al. demonstrated that patients with PE had a higher density of dNK cells in a resting, immature state. The high density of dNK cells affects their maturation and trafficking, leading to increased inflammatory cytokines and inhibiting trophoblast migration and invasion of the spiral arteries [81]. Controversies exist about the presence and causative role of dNK cells in the pathogenesis of PE and many have studies explored their functions rather than their number [82]. Research has shown that different types of immune cells, such as T cells, B cells, natural killer cells, and macrophages, are involved in the immune imbalance at the maternal–fetal interface in pre-eclampsia. PE is characterized by changes in both the quantity and quality of immune cell responses, both systemic and locally [83]. In healthy pregnancy, tolerance towards the fetus is granted by an inflammatory state. In PE patients, chronic inflammation is sustained by CD4 + T cells and pro-inflammatory cytokines, suppressing anti-inflammatory factors such as regulatory T cells (Treg) and IL-10. In an elegant study by Reeve et al., the authors demonstrated that CD4 + T cells isolated from PE patients can cause the formation of autoantibody to angiotensin II type 1 receptor, which may lead to increased activation of the RAAS and upregulation of angiotensin-converting enzyme 2 (ACE-2) [84].

#### 2.1.5. RAAS

The pathogenesis of PE also involves the renin–angiotensin pathway, particularly its alterations. This hormonal pathway regulates blood pressure and fluid and electrolytic balance. Renin is secreted into the bloodstream after a reduction of the renal blood flow. This protein can convert the angiotensinogen in angiotensin I, which is immediately converted to angiotensin II (angII) by ACE. AngII has a vasoconstrictive power and stimulates the release of aldosterone, which increases the resorption of ions and liquids, causing an increase in blood pressure. This mechanism is maintained by the circulating estrogen produced by the growing placenta, which enhances angII synthesis in the liver [85]. Confirmations of the involvement of this pathway in PE pathogenesis are found in several studies that show enhanced angII sensitivity of the adrenal cortex and vascular system during and before the onset of PE [85]. Renal tubular injury, derived by flt-1 inhibition of VEGF, caused an increased tubular permeability to most large-molecular-weight proteins, such as albumin, transferrin, and hemoglobin. High flt-1 levels inhibit podocyte-specific VEGF, disturbing the glomerular filtration barrier and forming fenestrae, contributing to proteinuria [86].

### 2.2. Histological Hallmarks: The Elementary Lesions

When the remodeling of the spiral arteries is defective, it leads to the atherosis of maternal radial arteries and placental vascular insults such as placental infarcts [87]. The histological hallmarks of PE are gathered into the maternal vascular malperfusion (MVM) category, as defined in the Amsterdam consensus [88]. The exact etiology of MVM is not well understood, but alterations in the normal remodeling process of the uterine vasculature during pregnancy are central to the pathologic process [89]. The defective transformation of the myometrial segments of the spiral arterioles at the junctional zone can give rise to lesions typical of placental MVM [90]. All these alterations cause hypoxia and, consequently, hypoxia-related injury. Gross findings of MVM are placental hypoplasia, placental infarction, and retroplacental hemorrhage. Microscopic lesions typical of MVM are distal villous hypoplasia and accelerated villous maturation. A placenta delivered from a patient with PE may exhibit histological changes, some of which are almost diagnostic of poor fetal–maternal interface interactions [91].

The elementary lesions of PE are listed below.

#### Decidual Arteriopathy

**Figure 2:**
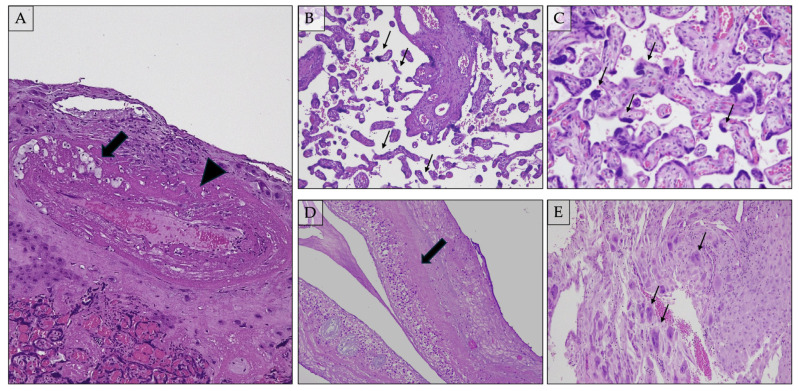
(**A**) Acute atherosis: fibrinoid necrosis (arrowhead) of the spiral arterial wall. Lipid-laden macrophages (arrow) are enclosed in the eosinophilic amorphous material, which surrounds the vessel (H&E, original magnification 50×); (**B**) distal villous hypoplasia; terminal villi have a “pencil-like” appearance (arrows) (H&E, original magnification 100×); (**C**) Tenney–Parker change (syncytial knots—arrows) (H&E, original magnification 200×); (**D**) deciduo-chorial necrosis (arrow) in the chorial membrane (H&E, original magnification 100×); (**E**) decidual multinucleated giant-cells (extravillous trophoblast—arrows) (H&E, original magnification 100×).

Decidual arteriopathy is a pathological change in decidual spiral arteries and can significantly affect fetal development. As previously described, the syncytiotrophoblast erodes the maternal spiral arteries during placentation to reduce resistance in blood flow. This process enables a low-pressure, high-flow blood connection between the mother and fetus, ensuring the fetus receives proper nutrition. The decidual spiral arteries play a crucial role in pregnancy by being involved in implantation and placentation. These arteries are invaded by the placental extravillous cytotrophoblast cells (EVCTs), which remodel the vessels by destroying the muscle layer and replacing the endothelial lining with a pseudo-endothelium of fetal origin. This transformation occurs between the 8th and 10th week of gestation [92,93]. In placentas destined to develop PE, cytotrophoblasts fail to transform from the proliferative epithelial subtype to the invasive endothelial subtype, which causes incomplete remodeling of the spiral artery. The narrow spiral arteries are prone to atherosis, characterized by lipid-laden macrophages within the lumen, fibrinoid necrosis of the arterial wall, and a mononuclear perivascular infiltrate (Figure 2A) [88,94]. This process is critical for the establishment of the definitive uteroplacental circulation. Inadequate vascular remodeling of the maternal spiral artery is histologically recognizable. Decidual arteriopathy is also an essential histopathological finding associated with PE. Decidual arteriopathy includes different histological aspects: acute atherosis and fibrinoid necrosis, mural hypertrophy and chronic perivasculitis, incomplete/absent physiological spiral artery remodeling, arterial thrombosis, and persistence of intramural endovascular trophoblast in the third trimester [88].

### 2.3. Acute Atherosis and Fibrinoid Necrosis

Uteroplacental acute atherosis was first described in 1945 by Hertig [95]. Acute atherosis is an uteroplacental spiral artery lesion in which the arterioles are affected by fibrinoid necrosis, a dense, often thickened, eosinophilic material beneath the endothelium that sometimes encloses lipid-laden macrophages. Acute atherosis is prevalent in PE and other obstetric syndromes such as FGR. Causal factors and effects of acute atherosis remain uncertain [96]. Acute atherosis shares morphological features with early atherosclerotic lesions, which is well recognized as an inflammatory arterial wall disease [97]. Both lesions present with increased intimal macrophages, lipid-laden foam cells, lipoprotein(a) throughout the vessel walls, and extracellular droplets of lipid [98], but acute atherosis is a uterine-restricted lesion [99]. Acute atherosis lesions are often found downstream of spiral arteries that have not been adequately remodeled, indicating a connection between the formation of acute atherosis and poor remodeling [100]. The lesions can be found in the basal plate and the decidua parietalis.

### 2.4. Mural Hypertrophy and Chronic Perivasculitis

According to Redline et al.’s definition, mural hypertrophy defines a thickening of the arteriole’s wall with a mean wall diameter of greater than 30% of the mean circumference of the arteriole, independently by the stratum that thickened (medial or subendothelial hyperplasia, hypertrophy, or matrix deposition) [101]. Mural hypertrophy is often associated with a mild, moderate perivascular lymphocytic infiltrate [88].

### 2.5. Incomplete/Absent Physiological Spiral Artery Remodeling

Vascular remodeling is a general term that includes all the structural changes in blood vessels involving cell proliferation and death, cell migration, and extracellular matrix modifications. The process of remodeling maternal spiral arteries is complex and not fully understood. In PE, the trophoblast invasion of the spiral artery can be incomplete or, in extreme forms, totally absent, resulting in the retention of a smooth muscle wall.

### 2.6. Arterial Thrombosis

Arterial thrombosis is caused by the presence of thrombi, made of fibrin, in basal decidual vessels [102].

### 2.7. Persistence of Intramural Endovascular Trophoblast

The persistence of intramural endovascular trophoblast refers to plugs of extravillous trophoblast that fail to disappear during the third trimester of pregnancy [103].

#### 2.7.1. Distal Villous Hypoplasia (Figure 2B)

Distal villous hypoplasia (DVH) is defined as a scarcity of terminal villi with an apparent increase in intervillous space [104]. DVH is a lesion caused by post-placental hypoxia, with non-branching angiogenesis leading to thin, elongated villi with limited ramification. The narrow, straight, unbranched villi take up less space than typically branching villi, resulting in an abnormally open maternal blood space [105]. DVH is not necessarily homogeneous throughout the placental tissue and may be diffuse or patchy. The diagnosis evaluates the lower two-thirds, avoiding the subchorionic plate (where villi are physiologically thinner) [106]. Distal villous hypoplasia is associated with early onset fetal growth restriction with absent or reversed end-diastolic flow of the umbilical artery. The villous tree undergoes changes in morphology and components, collectively called villous maturation [107]. Different villous types, such as stem villi, intermediate villi, and terminal villi, are characteristic of each particular stage of placental development. The percentage of each type identified in a placental section can help assess the degree of villous maturation.

#### 2.7.2. Accelerated Villous Maturation and Increased Syncytial Knots (i.e., Tenney–Parker Change) (Figure 2C)

Villous maturation is not generally uniform across the placenta, likely due to the differential flow through the spiral arteries [105]. In particular, accelerated villous maturation is defined as the presence of small or short (hypoplastic) hypermature villi for the gestational period, usually accompanied by an increase in syncytial knots [107]. The accelerated villous maturation mechanism is interpreted as a compensatory increased villous ramification and fetal vessel proliferation associated with high-burden maternal vascular malperfusion lesions (defined as two or more lesions of maternal vascular malperfusion). The proposed pathogenesis of accelerated villous maturation is defective remodeling of the spiral arteries [104], which leads to malperfusion of the placenta, causing loss of function through oxidative and endoplasmic reticulum stress, decreased surface area, damage to the syncytiotrophoblast, and hypoxemia [108]. Accelerated maturation is often accompanied by increased syncytial node formation. Syncytiotrophoblast, a multinucleated, terminally differentiated syncytium, acts as the endothelium for the placenta villous tree, physically separating maternal blood from fetal blood and other fetal tissues [109]. Syncytiotrophoblast nuclei increase ninefold from the 13th week of gestation to term [110]. Most nuclei are dispersed within the syncytioplasm, but others can form aggregates known as syncytial knots, which expand above the villous surface [109]. The nuclei within true syncytial knots display highly condensed chromatin dispersed throughout the nucleus or in a dense peripheral ring [109]. Normally, knots are rarely seen before the 20th week of gestation; their frequency increases as pregnancy proceeds and becomes particularly numerous in postmature placentas [111]. The increased number of syncytial knots is an actual alteration of villous morphology, and it derives from aggregates of syncytiotrophoblast nuclei on the villous surface. This alteration is also termed Tenney–Parker change [105]. The association of increased syncytial knots in patients with complicated pregnancies, for example, because of PE, is well known [112]. Syncytial knots microscopically consist of clusters of at least five syncytiotrophoblast nuclei that bulge above the normal villous surface. These nuclei are generally small and dark because of the elevated content of highly condensed chromatin.

#### 2.7.3. Basal Plate Lamina Necrosis (Figure 2D)

Laminar necrosis of placental membranes consist of a diffuse band of coagulative necrosis at the choriodecidual interface of the placental membranes, mixed with karyorrhectic debris [113,114]. Little is known about this type of necrosis, but it is thought that decreased uteroplacental perfusion (hypoxia) is a cause of basal plate laminar necrosis. The same kind of necrosis, with a band aspect, is found at the amniochorionic interface and, in this case, is known as laminar necrosis of placental membranes. Laminar necrosis is more frequently decidual or mixed rather than purely chorionic [114].

#### 2.7.4. Multinucleated Giant Cells in Decidua Basalis (Figure 2E)

At the beginning of placentation, immediately after attachment, the trophoblast cell layer of the blastocyst proliferates rapidly and differentiates into an inner cytotrophoblastic layer and an outer multinucleated syncytiotrophoblastic mass. The syncytiotrophoblast extends into the endometrial epithelium and invades the connective tissue. Interstitial trophoblast cells become multinucleated and more rounded, forming placental bed giant cells as they move deeper into the decidua [1]. These cells are regarded as the terminally differentiated end-point of the extravillous pathway. In the early phases of placentation, multinucleated placental-bed giant cells (MTGC) are the hallmark of trophoblast invasiveness, found in the inner third of the myometrium under the implantation site [115]. In later stages of pregnancy, MTGC is regarded as a feature of maternal vascular underperfusion in PE [116]. Placentas from pre-eclamptic pregnancies tend to be small and have abnormally superficial uterine implantation, a defect that may be caused by inappropriate expression of integrin substrate adhesion molecules. This could be one aspect of a generalized maturation defect that leads to trophoblast excess via the accumulation of immature trophoblast in superficial regions of the implantation site [117].

#### 2.7.5. Infarcts

Placental circulation brings into proximity two discreet circulatory systems, the fetal and maternal placental blood flow, separated by a selective anatomic barrier [118]. The circulation within the placental parenchyma brings oxygen and nutrients while removing waste products. In case there are any anomalies in circulation, there may be compensatory or harmful effects on placenta parenchyma. A placental infarct is an area of ischemic coagulative necrosis of the placental parenchyma, which results from the flow restriction by a narrowing of thrombotic occlusion of maternal myometrial and decidual arterioles. An area of infarction comes from near-complete or complete obstruction to circulation in a localized area, so its pathogenesis is similar to infarction in other organs, that is, a rapid loss of arterial blood supply [119]. This area is characterized by the collapse of the intervillous space and villous ischemic necrosis. Placental infarcts are macroscopically evident focal parenchymal lesions in which microscopy shows necrosis and approximation of villi [1]. Infarcts are considered a marker of MVM. Placental infarcts, particularly multiple and larger infarcts, are historically known to be commonly observed in association with severe early-onset PE and intra-uterine growth retardation in pregnancies that are unlikely to reach term [116]. Placental infarction is a commonly observed gross abnormality during pathological examination of the placenta. Infarcts have been detected in 10.9% of term placentas that come from pregnancies without fetal growth restriction or maternal hypertension [119]. Although placental infarcts are common in the latter stages of pregnancy, in the first or second trimester, they are considered abnormal. Infarcts associated with maternal hypertension and fetal growth restriction are usually multiple and centrally located. During the examination of the placenta, an infarct is identified as an area of discoloration in the tissue. This area becomes paler due to the loss of blood flow to the villi, loss of red cells in the space between the villi, and the buildup of fibrinoid material around the villi. The infarct can be found anywhere in the placenta, either centrally or marginally, and can be single or multiple. An infarct size can range from 2–3 cm, and in some cases, infarcts can be connected. Sometimes, a central hemorrhage is present in the infarct area, referred to as rounded intraplacental hematomas or infarction hematomas. The histopathological features of acute placental infarction include the collapse of the intervillous space and degenerative changes in the villous trophoblasts. These changes are characterized by the loss of nuclear basophilic staining and the loss of nuclear detail. Additionally, fetal vessels lining large infarcts may exhibit thrombotic changes [74,120]

#### 2.7.6. Placental Abruptio

Placental abruption, defined as the premature separation of a normally implanted placenta, complicates approximately 1% of deliveries and may also be implicated in up to 10% of preterm births [121]. Premature separation of a portion of the placenta from the uterine surface may cause bleeding from decidual vessels and result in the formation of a retroplacental hemorrhage (RPH). After childbirth, the detachment of the placenta normally takes place when the empty uterus vigorously contracts and compresses decidual vessels to stop the physiological bleeding. Premature separation, on the contrary, leads to bleeding because the uterus, still bearing the fetus and the amniotic fluid, cannot contract with a strength sufficient to compress the decidual vessels and stop the bleeding. “Placental abruption” is the obstetrical diagnosis of the separation of a normally implanted placenta from the uterine wall, which causes symptoms like vaginal hemorrhage, uterine pain, and/or fetal distress. If blood cannot flow out of the uterine cavity, it will form a retroplacental hematoma (blood clot) [122]. A retroplacental hemorrhage arises in the decidua under the functional placenta. Dommisse et al. described a small case series of placental bed biopsies in 12 women with abruption that demonstrated a lack of adequate trophoblastic invasion in seven (58%) of them [123]. A prospective cohort study has found an association between impaired uteroplacental blood flow at 20–24 weeks and the subsequent development of placental abruption [124].

#### 2.7.7. Increased Perivillous Fibrin

Fibrin is the shorthand term for a substance more appropriately called fibrinoid. Perivillous fibrinoid material is a mixture of blood proteins, including fibrin, fibrinogen, fibronectin, basement membrane collagen, laminin, and major essential proteins. This fibrinoid material occupies the perivillous space, and an increase in that is designated as a pathologic process. Most placentas contain perivillous fibrin, which increases with gestational age [125]. The fibrinoid material in the placenta is shown to be mainly derived from maternal circulation (as a by-product of endothelial damage), which is called fibrin-type fibrinoid.

#### 2.7.8. Extravillous Trophoblastic Cysts

Extravillous trophoblast cysts can be detected in the subchorionic region or within the septa. Their pathogenesis is strictly correlated to hypoxia, which is essential in their development because cytotrophoblast proliferates in response to hypoxia. The incidence of extravillous trophoblast cysts is increased in cases of PE and maternal diabetes. If these cysts are located under the chorionic plate, they are named subchorionic cysts; if they are located within the placental septa, they are named septal cysts. The latter are invaginations of the decidual basal plate that anchor the chorionic plate onto the basal plate. Some extravillous trophoblast cysts are grossly visible, but most are seen microscopically. The cyst wall is composed of an irregular layer of extravillous trophoblastic cells, filled with an amorphous eosinophilic proteinaceous fluid [88,120].

## 3. Conclusions

PE is a serious and late complication of pregnancy and is one of the leading causes of maternal and fetal illness and death worldwide. The symptoms experienced by the mother are due to the placenta not implanting and developing properly during the early phase of the pregnancy. It seems that an important factor in the development of pre-eclampsia is an imbalance in the growth of blood vessels in the lining of the uterus during implantation. This imbalance includes an overproduction of anti-angiogenic factors such as sFlt-1 and a reduction in angiogenic factors like VEGF. The presence of sFlt-1 in the mother’s blood may help identify pregnancies at risk for pre-eclampsia. During the examination of the placenta of pre=eclampsia patients, the corresponding histological features can be identified and classified accurately.

## 4. Future Directions

In recent studies, some authors describe an Alzheimer-like pattern of impaired autophagy and proteinopathy in the placenta of PE patients, which constitutes a novel molecular rationale for impaired autophagy and proteinopathy in patients with PE. The recognition and targeting of these pathological pathways may lead to a novel therapeutic strategy for PE [126].

## Data Availability

Data are contained within the article.
